# Development and Validation of the Portuguese Transcultural Nursing Leadership Questionnaire (QLTE-PT)

**DOI:** 10.1155/2024/5750265

**Published:** 2024-06-24

**Authors:** Gisela Teixeira, Filomena Gaspar, Pedro Lucas

**Affiliations:** Nursing Research Innovation and Development Centre of Lisbon (CIDNUR), Nursing School of Lisbon, Avenida Prof. Egas Moniz, Lisboa 1600-069, Portugal

## Abstract

**Introduction:**

This study introduces the Portuguese Transcultural Nursing Leadership Questionnaire (QLTE-PT), a pioneering instrument designed to assess leadership behaviours in multicultural nursing work environments, addressing gaps in current leadership assessment tools.

**Aim:**

This study aimed to develop and validate the Portuguese Transcultural Nursing Leadership Questionnaire (QLTE-PT).

**Methods:**

It was conducted as a sequential exploratory mixed-method study, integrating DeVellis's steps for instrument development. Items were formulated based on a literature review and a focus group study, and the content validity was evaluated by a panel of experts. A methodological approach involving nurses registered in the Portuguese Order of Nurses with leadership experience in multicultural nursing work environments was employed to further conduct an exploratory and a confirmatory factor analysis to assess the instrument's structure and psychometric properties.

**Results:**

One hundred forty-five items were initially generated, of which 39 were included in the QLTE-PT following content validity assessment by a panel of experts. EFA revealed a factor structure of 25 items loading on six factors, explaining 64% of the total variance. The overall Cronbach's *α* coefficient of the questionnaire was 0.90. This six-factor structure was tested by CFA, revealing a final model of 23 items and six factors, with a good quality of adjustment (CFI = 0.980, TLI = 0.976, SRMR = 0.078, and RMSEA = 0.070). Both convergent and discriminant validity were confirmed.

**Conclusions:**

The QLTE-PT demonstrates good psychometric properties and is suitable for assessing transcultural leadership behaviours of nurse managers and leaders in multicultural nursing work environments. *Implications for Nursing Management*. The QLTE-PT can assist nurse managers to improve their leadership behaviours, promote supportive working environments for their multicultural nursing staff, and improve the quality of care provided to patients from different cultural backgrounds.

## 1. Introduction

The interconnectedness and interdependence between nations, along with the exchange of people, goods, services, and information, have contributed to cultural diversity in workplaces, communities, and globally. This requires leaders who can guide multinational projects and lead people or groups from different cultural backgrounds in organizations, producing appropriate responses to their customers' needs with different cultures and expectations [1, 2]. Against this backdrop, the concept of transcultural leadership has gained prominence, advocating for the development of people and organizations equipped to address the challenges that arise from globalization, increased competition, and power asymmetries [3].

Defined as the process of articulating, implementing, and nurturing a global cultural vision and creating multicultural synergy [4], transcultural leadership is pivotal in various sectors, including healthcare. Within the nursing discipline, transcultural nursing leadership focuses on the culturally sensitive transformation journey. This involves tailoring behaviours, processes, and products to meet the cultural needs of both nurses and patients, challenging traditional paradigms, and guiding the delivery of culturally congruent care [5]. Such leadership is essential not only for delivering culturally congruent care to patients and achieving optimal health outcomes for all populations [6, 7] but also for effectively managing the challenges of culturally diverse nursing teams [5].

Cultural diversity among patients and healthcare professionals is a current scenario in Portuguese healthcare organizations [8]. Portugal hosts more than half a million of immigrants [9], who face several difficulties when accessing health services, such as discrimination, lack of information and knowledge on how the Portuguese healthcare system works, administrative barriers, and cultural and linguistic barriers that hinder communication with the employees and healthcare professionals of the National Health Service [10]. According to a study developed by Dias et al. [11], a majority of immigrants consider that having been in Portugal for a short time (85.1%), lacking knowledge about legal rights to health access (78.9%), being alone in Portugal (76.9%), having insufficient financial resources (75.6%), existence of complex bureaucratic procedures for health services access (75.2%), language differences (59.8%), absence of interpreters (49.5%), challenges in expressing symptoms and recognizing illness (46.9%), distrust towards health professionals (45.7%), and beliefs, religious, and cultural traditions (27.7%) are factors that condition their access and utilization of health services in Portugal.

In addition, recent data from the Portuguese Order of Nurses Ordem dos Enfermeiros [12] reveal that 1348 nurses registered in 2022 were from foreign countries. According to Silva and Fernandes [13], despite the reports of a good welcome, immigrant nurses report challenges and difficulties during their integration in Portugal. These difficulties and challenges are related to the recognition of academic degrees and the differences in roles, skills, autonomy, and hierarchy in professional relationships, working conditions/resources, and language, particularly differences in technical nursing terms between Portugal and their home countries. Foreign nurses in Portugal also identify experiences of discrimination from patients and colleagues and unequal treatment by their managers, such as discrediting their knowledge, exclusion from training, unbalanced work distribution, abuse of power, and no opportunities for professional development, specifically being a team leader or mentoring nursing students [13]. According to Primeau et al. [14], it is important that organizations ensure healthy work environments free of discrimination and with opportunities for this particular group of nurses to achieve their career goals.

The diversity and related challenges in Portuguese healthcare settings highlight the need for a nuanced understanding and effective management of cross-cultural dynamics, accentuating the importance of transcultural nursing leadership. Nurse managers are pivotal in this context, as they are responsible for promoting culturally congruent care to patients from different cultural backgrounds and developing favorable work environments for multicultural nursing teams [15]. The challenges they face in providing better support to their teams and developing effective leadership styles [16], especially when managing nurses from different cultures, are significant. This complexity is a growing concern in Portugal [17–21] and the complexity that cultural diversity adds to these environments must be recognized.

There are no studies or instruments to assess transcultural nursing leadership. The most commonly used tools to assess nursing leadership competencies are the Ambulance Nurse Competence scale, Leadership Practices Inventory, Clinical Leadership Needs Analysis Instrument, Cotter Preceptor Selection Instrument, Performance Evaluation Tool, Leadership and Management Inventory, Advanced Practice Nursing Competency Assessment Instrument, and Kuopio University Hospital Transformational Leadership Scale [22]. Given the absence of instruments assessing transcultural nursing leadership in multicultural settings, i.e., in healthcare settings where nurses from different cultural backgrounds work and where care is provided to culturally and linguistically diverse patients, this study aimed to do the following:

1. Develop the Portuguese Transcultural Nursing Leadership Questionnaire (QLTE-PT) specifically tailored to assess nurse leaders and managers' leadership behaviours in multicultural nursing environments in Portugal
2. Validate the QLTE-PT by assessing its internal consistency, confirming its construct validity through exploratory and confirmatory factor analyses, and establishing both its convergent and discriminant validity

## 2. Materials and Methods

A sequential exploratory mixed-method study was conducted in two stages [23]. The first stage aimed to develop the questionnaire. The second stage followed a quantitative study to analyze its psychometric properties.

The questionnaire development and validation process were based on DeVellis's [24] steps for instrument development which are as follows: (a) determine what is intended to be measured; (b) generate a set of items; (c) determine the measurement format of the instrument; (d) review the set of items formulated from a panel of experts; (e) consider the inclusion of validation items; (f) apply the items to a sample; (g) evaluate the items; and (h) optimize the dimensionality of the instrument.

### 2.1. Generate a Set of Items

Items were generated based on a scoping review and a focus group, which are both strategies to generate a pool of items for new instruments [25, 26].

#### 2.1.1. Scoping Review

The scoping review was conducted in accordance with JBI guidelines [27], aiming to map the personality traits, competencies, behaviours, and leadership styles of nurse leaders and managers impacting the outcomes of multicultural nursing teams. Papers published in English, Portuguese, and Spanish languages were searched through electronic databases, such as CINAHL, MEDLINE, Nursing & Allied Health Collection, MedicLatina, Psychology and Behavioural Sciences Collection, Wiley Online Library, and Scopus. We did not restrict the publication dates in our scoping review due to the lack of previous literature reviews on this topic. This approach enabled us to comprehensively access the best available evidence without temporal constraints. The search strategy comprised the following keywords: nursing leadership, leadership traits, competencies, behaviours, skills, and styles; multicultural nursing teams, and nurses' outcomes.

#### 2.1.2. Focus Group

A qualitative, exploratory focus group study was conducted to explore the strategies used by nurse managers in creating favorable work environments for multicultural teams and delivering culturally congruent care to diverse patients [28]. A convenience sample of five Portuguese nurses with experience leading multicultural nursing teams was recruited to participate in the study. A semistructured interview was performed with questions aimed at eliciting participants' insights on transcultural nursing leadership, effective management interventions for multicultural nursing teams, and strategies to improve culturally congruent care. Qualitative data were recorded and transcribed for content analysis.

Based on the results of both studies, a list of the most relevant nurse leaders and managers' behaviours in multicultural nursing work environments was produced. The items were revised to ensure appropriate wording and to remove duplicates. A first draft of the questionnaire was prepared.

### 2.2. Determine the Measurement Format of the Instrument

All items of the questionnaire were scored based on a 5-point Likert scale ((1) “never,” (2) “rarely,” (3) “sometimes,” (4) “frequently,” and (5) “always”), representing a continuum of the frequency of nurse leaders or managers' behaviours in multicultural nursing work environments. This choice is based on Boateng et al.'s [25] guidelines, which advocate that response scales with five to seven points have higher reliability than Likert-type response scales with less than five points.

### 2.3. Reviewing the Set of Items Formulated from a Panel of Experts

Once the items had been formulated, an assessment of the questionnaire's psychometric properties was conducted to analyze whether it was adequate and accurate in assessing transcultural nursing leadership, as well as to evaluate its validity and reliability [29]. This stage comprised two phases: content validity and construct validity.

Content validity is invaluable for the quality of a newly developed instrument [29]. Since there is no specific statistical test for this purpose, it is common to use a panel of experts to assess the set of items formulated and validate whether they accurately represent the construct measured by the new instrument [30].

The experts were recruited by convenience in a Portuguese association of nurse managers and leadership. Following the recommendations of Polit and Beck [26], it was defined that at least two rounds would be performed to determine the content validity of the items and the questionnaire as a whole. In the first round, each item was assessed regarding its degree of relevance, clarity, simplicity and ambiguity, on a scale of 1 (not relevant) to 4 (very relevant), according to the criteria described by Yaghmale [31]. Once assessed in each criterion, the content validity index of each item (I-CVI) was calculated based on the ratings assigned by the experts regarding its degree of relevance [26, 29].

Following the authors' recommendations, it was determined that items with a I-CVI of <0.78 would be excluded from the questionnaire [26, 29]. The items with a I-CVI of ≥0.78 but with values of <1 in the criteria of clarity, simplicity, and ambiguity, estimated according to the previous formula were reformulated. After this process of item deletion and reformulation, a second round of relevance assessment of the remaining and reformulated items was carried out. The I-CVI of each item was calculated and the overall content validity index of the questionnaire (Ave-CVI) was estimated from the average of the I-CVIs [29]. It was considered that a I-CVI ≥0.78 on each item and an Ave-CVI of ≥0.90 would be indicative of an excellent content validity [26]. The estimated value of Ave-CVI in the second round would determine the need for questionnaire reformulation and additional rounds.

### 2.4. Consider the Inclusion of Validation Items

Although it is possible to include additional scales that may provide information about the validity of the final questionnaire [24], it is recommended that researchers limit these efforts at this stage of developing a new instrument. According to Worthington and Whittaker [32], it is advisable to keep the overall length of the questionnaire as short as possible and directly related to the central aim of the study. Worthington and Whittaker [32] also argue that there is a potential risk that items from other scales may interact with the items designed for the new instrument, interfering with its development process. Therefore, it is important to avoid influencing the items' responses during the initial phase of instrument development, thus limiting the use of additional measures [32]. Understanding the risks highlighted by Worthington and Whittaker [32], it was decided to not include validation items as recommended by DeVellis [24].

### 2.5. Evaluating and Refining the Instrument

Following the establishment of content validity, a quantitative and cross-sectional study was conducted to identify the core dimensions of transcultural nursing leadership and to confirm the reliability of the instrument. For this purpose, an exploratory factor analysis (EFA) was performed followed by a confirmatory factor analysis (CFA).

Nurses registered in the Portuguese Order of Nurses in the categories of nurse manager, nurse specialist, or nurse, with current or past leadership experience in multicultural nursing work environments were invited to participate in this study. Nurses without leadership experience in multicultural nursing work environments were excluded. The sample size was based on guidelines recommending five to ten participants for each questionnaire item to ensure a robust analysis in EFA and CFA [33].

During EFA, the correlations between items were assessed using the Kaiser–Meyer–Olkin (KMO) measure to ensure that they were suitable for factor analysis. The analysis was then performed using an appropriate estimator for ordinal data (WLSMV), robust to deviations from normal distribution [34]. For the extraction of factors in our EFA, we employed the principal component as the method to identify the initial structure of latent factors, followed by an oblique rotation to explore the relationships between these factors. The number of latent factors identified considered both the eigenvalues greater than 1 and the scree plot. We then retained factors explaining a significant portion of the total variance and removed individual items with factor loadings below 0.50.

The instrument was refined by repeating EFA, ensuring that each factor was reliable and represented by at least two items. The quality of the factor structure was assessed using the root mean square residual (RMSR) index.

Confirmatory factor analysis was performed to assess the validity of the factor structure identified in EFA. It analyzed the existence of outliers by analyzing the square distance of Mahalanobis (*D*^2^). The normality of the variables was assessed by the uni- and multivariate asymmetry (|Sk| < 3) and kurtosis (|Ku| < 10) coefficients [35]. The quality of the model was assessed through the indices proposed by Brown [36] and Marôco [35]: CFI (<0.8: bad fit), TLI ((0.9; 0.95): good fit), SRMR (≤0.08: good fit), and RMSEA (>0.08–0.10: unacceptable; (0.05; 0.08): acceptable; and ≤0.05: very good). Necessary adjustments were made based on modification indices greater than 11 and theoretical justification, as recommended by Marôco [35].

Finally, item reliability was assessed based on the proportion of variance accounted for by the latent factor, with a target value of 0.25 or higher [35]. The overall instrument's reliability, as well as that of individual factors, was determined using Cronbach's alpha, setting a minimum acceptable level of 0.70 [37]. Construct validity was established through analyses of convergent and discriminant validity [30, 35]. Missing values were handled with the pairwise method in both EFA and CFA.

All statistical analyses were conducted in RStudio (© 2009–2022 RStudio, PBC) using packages “polycor,” “psych,” “lavaan,” and “lavaanPlot.”

### 2.6. Ethical Considerations

This study was first approved by the Ethics Committee of the Nursing School of Lisbon (approval no. 216/2022/CE), as part of the doctoral research project. Authorization and support were requested from a Portuguese association of nurse managers and leadership to recruit nurses to integrate the panel of experts to perform the content validity of the questionnaire. Once the content validity phase was completed, authorization and support were also requested from the Portuguese Order of Nurses to disseminate the study to its members and invite those with the experience of leadership in multicultural nursing work environments to complete the questionnaire online. All participants approved their participation through an electronic consent form, without which they could not proceed to data collection. All participants were informed that they could withdraw from the study at any moment. Anonymity and data confidentiality were guaranteed.

## 3. Results

### 3.1. Generation of Items

A total of 115 and 63 items were formulated from the scoping review and the focus group study, respectively. After removing duplicates and similar items, 145 items composed the initial version of the QLTE-PT.

### 3.2. Content Validity

A total of six members of the Portuguese association of nurse leaders and managers participated in the panel of experts, meeting the minimum number of experts recommended to assess the content validity of an instrument [29]. Half of the participants were female, with a mean age of 53.8 years (SD = 7.7) and 31.7 years of professional experience (SD = 7.4). More than 50% held a master's degree or PhD degree, 16.7% were nurse specialists, and 16.7% had a bachelor's degree. About 67% were nurse managers. All experts of the panel stated that they worked or had worked with professionals from different cultural backgrounds, but none of them were or had been emigrants. None of the participants had education in multiculturalism.

Of the 145 items rated by the experts regarding their degree of relevance, 98.6% (*n* = 143) had a I-CVI ≥ of 0.78. Two items were excluded from the questionnaire due to I-CVI = 0.67. Only two participants wrote comments/suggestions for improvement, which were “simplify some items.” In some cases, it may even be necessary to “split one item into two” and “some questions were too long, which makes it difficult to answer them.” However, these two participants did not specify items for these actions. Due to the lack of specific guidance on which items to modify, a conservative approach proceeded in the revision process. The original structure of the items was maintained, focusing instead on improving items' clarity, simplicity, and reducing ambiguity. Fifty-one items, which had clarity, simplicity, or ambiguity indices below 1, were rephrased. These revised items, along with the others, constituted a set of 143 items that underwent a second round of evaluation. Five of the six experts from the previous round participated in the second round. Of the 143 items, 94.4% (*n* = 135) obtained a I-CVI of ≥0.78 and eight were eliminated due to a I-CVI of <0.78. No comments or suggestions for improvement were made in the second round. The questionnaire showed an Ave-CVI of 0.96. As a strategy to obtain a more parsimonious set of items, it was decided to consider the items' Content Validity Ratios (CVRs).

**Figure 1:**
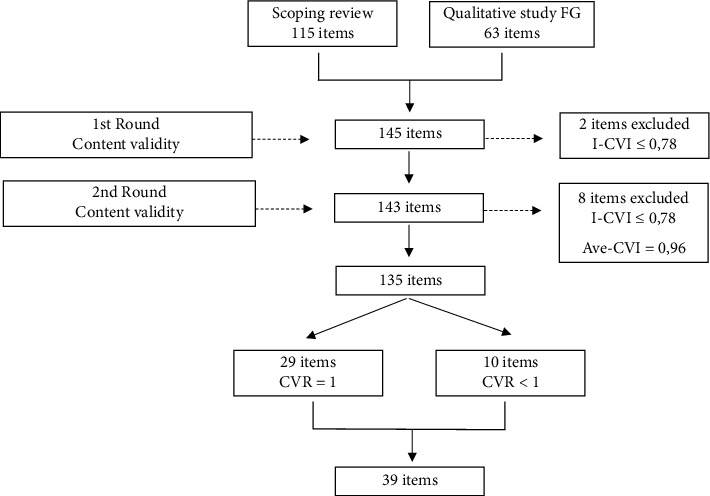
Process of items' generation, inclusion, and exclusion from the QLTE-PT.

Twenty-nine items were identified as essential/very relevant with CVR = 1. It was decided to retain 10 additional items, despite having a CVR of <1, given their relevance highlighted in the literature. For instance, item “I adapt my leadership style according to the expectations, values, habits, beliefs, and cultures of the members of the nursing team member” was aligned with established theoretical frameworks [3, 38] and empirical studies [5, 28] within the field that underscore their significance in assessing transcultural leadership. These decisions resulted in a total of 39 items included in the QLTE-PT, categorized into seven hypothetical dimensions: impartiality and nurse manager's ability to adapt, understand, accept, and respond to cultural differences; professional and sociocultural integration of immigrant nurses; standardization of nursing practice; supporting professional development; managing problems in multicultural teams; intercultural communication; and culturally congruent services and care.  Figure 1 summarizes the process of item generation, inclusion, and exclusion from the QLTE-PT.

### 3.3. Reliability of QLTE-PT

#### 3.3.1. Sample Characteristics

Four hundred and sixty-three nurses answered the questionnaire between November 2022 and March 2023, among whom 79.3% were female and 21.7% were male. The average age of the participants was 41.3 years (SD = 10.6), and the mean length of professional nursing experience was 18.2 years (SD = 10.6). More than 20% reported working or having worked abroad, namely, in Belgium, Brazil, Cape Verde, France, Germany, Guinea-Bissau, Indonesia, Oman, São Tomé and Príncipe, Saudi Arabia, Switzerland, Timor-Leste, and the United Kingdom. Data about education level, professional category, work unit, exposure to other cultures and in which context, training in multiculturalism, and international professional experience are comprehensively presented in Table 1.

#### 3.3.2. Exploratory Factor Analysis

EFA was performed to identify the relational structure among the 39 items of the QLTE-PT. The findings of the preliminary factor analysis showed a KMO value of 0.85, which indicates the adequacy of the instrument's items for factor analysis. The factorability was confirmed for the 39 items by Bartlett's test of sphericity (*X*^2^=1370.5; *p* < 0.001).

According to the criterion of eigenvalue greater than 1 and in line with the scree plot, the relational structure of the QLTE-PT would be explained by the nine latent factors explaining 73.2% of the variance. This initial factor structure of the questionnaire resulted in a problematic factor with only one item (item A32). Subsequent attempts to refine the structure by extracting eight factors still yielded another one-item factor (item A17). Even when reducing the factors to seven, the internal consistency of the two factors fell below the recommended threshold of 0.70 (*α* = 0.62 and *α* = 0.66). To improve the questionnaire's psychometric properties, we conducted further analyses, ultimately settling on a factor structure with six factors, which provided a more coherent and interpretable model. The factor structure comprising six factors was successfully derived, explaining a total variance of 64%. Fourteen items were removed due to factor loadings below 0.50. The six retained factors were found to aptly describe the correlational structure between the items, and the factor structure exhibited a good quality of adjustment (RMSR = 0.059). The overall internal consistency of the questionnaire was demonstrated to be robust (*α* = 0.90). In addition, each of the six factors retained in the factor analysis also showed a good internal consistency (*α* ≥ 0.69). These results affirm the reliability of the instrument both as a whole and in its individual factors.  Table 2 provides a comprehensive summary of the factor loadings, communalities, percentage of explained variance, overall internal consistency, and individual factors' internal consistency.

#### 3.3.3. Confirmatory Factor Analysis

The first CFA revealed an initial poor quality of adjustment (CFI = 0.961, TLI = 0.955, SRMR = 0.089, and RMSEA = 0.091). To enhance the model, outlier observations were removed. Items A24 and A25, which exhibited modification indices suggesting saturation in at least two other factors, were also removed from the model. Furthermore, we correlated the measurement errors of items with higher modification indices (A34-A35, A3-A4, A35-A6, A5-A6, A35-A31, and A15-A8) to improve the model quality. Following these refinements, the model demonstrated good fit indices (CFI = 0.980, TLI = 0.976, SRMR = 0.078, and RMSEA = 0.070) and good internal consistency, providing robust support for the factor validity of the QLTE-PT.

All items had standardized factor loadings greater than 0.50 and individual reliability (*λ*_*ij*_ ≥ 0.25). It was observed that the average variance extracted (AVE) supported all factors' convergent validity. The discriminant validity of the factors was evaluated by comparing the AVE with the squared correlations between the factors (Table 3). It was found that each of the factors had an AVE greater than the square of its correlation with the other factors, which supported that all factors had a discriminant validity.

**Figure 2:**
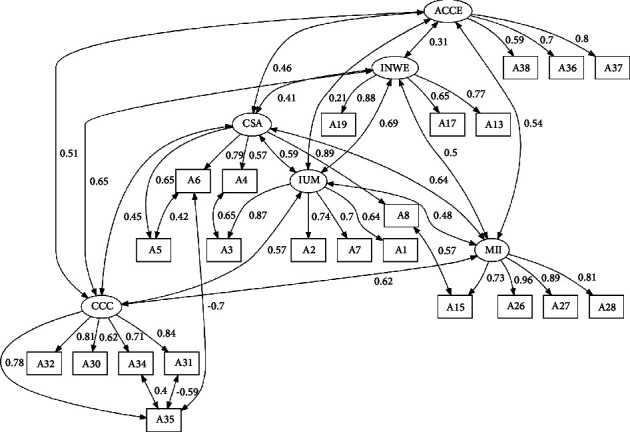
Six-factor model of QLTE-PT (CFI = 0.980, TLI = 0.976, SRMR = 0.078, and RMSEA = 0.070). Standardized loads and covariances.

The first factor was designated as “supporting culturally congruent care” (CCC), the second as “managing intercultural issues” (MII), the third as “inclusive and unbiased management” (IUM), the fourth as “cultural sensitivity and adaptation” (CSA), the fifth factor as “integration in the nursing work environment” (INWE), and the last factor was designated as “adjusting care to cultural expectations” (ACCE).  Figure 2 provides a visual representation of the final six-factor model of QLTE-PT.

## 4. Discussion

The development and validation of the Portuguese Transcultural Nursing Leadership Questionnaire (QLTE-PT) fills the gap in assessment tools for transcultural nursing leadership. It revealed a six-factor structure and 23 items that robustly encapsulate essential aspects of transcultural nursing leadership. These factors include supporting culturally congruent care, managing intercultural issues, inclusive and unbiased management, cultural sensitivity and adaptation, integration in the nursing work environment, and adjusting care to cultural expectations. The instrument demonstrates strong internal consistency, with an overall Cronbach's alpha value of 0.90, which is higher than the expected *α* and equal or higher than 0.70 for new instruments [37]. It explains a total variance of 64%, which is also higher than the minimum expected for social sciences [33], indicating its effectiveness in capturing the complexity of transcultural nursing leadership in multicultural nursing work environments.

During the stage of the questionnaire's development, 135 items obtained a I-CVI of ≥0.78 and the questionnaire showed an Ave-CVI of 0.96, meeting the required criterion of Ave-CVI of ≥0.90 [26, 29]. Although the criteria for an excellent content validity were assured, the high dimensionality of the questionnaire in its initial development phase raised concerns. In the early stages of questionnaire design, it should not be so long that it reduces the likelihood of potential participants answering or completing all the items, nor too short that it fails to cover all aspects of the construct it is intended to measure [32]. Such imbalances pose risks to the internal consistency of the construct and can affect the relationships and correlations among items within specific factors or domains [29]. As a strategy to obtain a more parsimonious set of items, it was decided to consider also the items' CVR as proposed by Lawshe, cited by Almanasreh et al. [29], which reduced the number of items from 135 to 29.

The inclusion of 10 additional items with a CVR of <1 proved to be a significant step in the development of the QLTE-PT. Only two of these items were dropped during the EFA due to factor loadings below 0.50, which underscores the importance and relevance of the remaining eight items, as they contribute meaningful dimensions to the overall construct despite their initial lower CVR scores.

The first factor, “supporting culturally congruent care,” showed good internal consistency, as evidenced by a high Cronbach's alpha value (*α* = 0.81). This indicates that the items within this factor reliably measure the intended construct. The factor accounted for a significant portion of the total variance (13.9%), demonstrating its substantial contribution to the overall model. This factor comprises five items that encapsulate the actions taken by nurse leaders and managers to assist nurses in enhancing the quality of care they provide to patients from different cultural backgrounds. Examples of such actions include “I encourage immigrant nurses to take a course on the local language and facilitate their schedules to be able to attend it,” “I conduct trainings that enable nurses to provide culturally congruent nursing care,” and “I provide nurses with pamphlets and handbooks related to the delivery of culturally congruent nursing care.” These items align closely with the established strategies to improve culturally congruent care in nursing literature, which supports the convergent validity of this factor. According to Russell [7], a transcultural nurse leader engages nurses, nursing students, and other healthcare professionals in a learning process of what it means to deliver culturally congruent care to culturally diverse populations. Providing written documentation on culturally congruent care [39], introducing transcultural education in services [40] and encouraging nurses to attend culture-related training sessions and activities, and ensuring their coverage during their absence from the shift [41] are a few examples of how this learning process may be conducted that support this factor. This factor includes also an item related to the nurse leader or manager's improvement to communicate effectively with nurses of different nationalities and cultures, which is supported by the literature about the importance of engaging in ongoing training that enhances the skills related to culture and diversity in the workplace [41]. The discriminant validity of this factor is supported by its distinctiveness from other factors in the model. The relatively low correlations with other factors indicate that it measures a unique aspect of transcultural nursing leadership, separate from other leadership dimensions captured in the QLTE-PT.

The second factor “managing intercultural issues” contains four items, for instance, “I investigate events related to verbal or physical abuse, discriminatory behaviour, and harassment, and implement measures that reduce the risk of occurrence.” It comprises nurse leaders and managers' attitudes and actions to prevent, resolve, and mitigate problems arising from cultural differences between nurses. This factor demonstrated a good internal consistency (*α* = 0.79), reflecting the coherence of its four items in assessing leadership approaches to intercultural challenges. The factor's significant contribution to the total variance (13.6%) underscores its importance in the overall construct of transcultural nursing leadership. Convergent validity for this factor is supported by literature emphasizing the critical role of nurse leaders in addressing and resolving cultural conflicts and fostering a harmonious work environment. For instance, Munkejord [42] argues that healthcare managers can contribute to challenging the ethnic pyramid often identified in culturally diverse institutions by implementing diversity-sensitive measures. Encouraging and arranging dialogue and collaboration among staff are one of those measures that lead to better relationships and reduce ethically based discrimination [42]. Nurses from ethnic minorities need to feel supported in the nonthreatening work environment. They also need to know that corrective actions will be implemented if the behaviour of an employee or group of employees is considered harmful [43]. The distinctiveness of this factor from others in the questionnaire, as shown by its discriminant validity, highlights its unique role in transcultural nursing leadership.

Factor 3 was labelled as “inclusive and unbiased management” as it concerns the degree to which the decision-making process of the nurse leader or manager deviates from favoring some nurses. It demonstrated a significant internal consistency (*α* = 0.76), which suggests that the item effectively captures the essence of unbiased and inclusive decision-making in transcultural nursing leadership. Factor 3 is composed offour items, including“I have a transparent policy in the unit regarding the method of organizing nursing care, schedules, holidays, annual appraisal, and promotions.” Convergent validity for this factor is evident in its alignment with existing literature on the importance of equity and impartiality in leadership, particularly within culturally diverse healthcare settings. Studies have highlighted that nurse managers who show favoritism, plan schedules, and vacations unequally among nurses of different cultural backgrounds, and promote nurses based on cultural criteria over merit, impact negatively on migrant nurses' job satisfaction, retention, and professional development [44, 45]. Discriminant validity is also well-established, with this factor distinctly measuring characteristics of leadership that are separate from others, as suggested by its low correlations with other factors in the QLTE-PT. This uniqueness is crucial in a transcultural context where unbiased and inclusive management is key to harmonizing a diverse workforce.

The fourth factor “cultural sensitivity and adaptation” is composed of four items aiming to describe nurse leaders and managers' sensitivity to cultural differences and how this sensitivity is reflected in their attitudes and behaviours towards nurses. The internal consistency of this factor (*α* = 0.76) confirms the reliability of the items in measuring cultural sensitivity and adaptation in nursing leadership. This factor's convergent validity is supported by the existing research, such as studies by Sharifi et al. [46], which emphasizes cultural sensitivity as an attribute of cultural competence in nursing, translating into valuing, respecting, and admiring cultural diversity and helping nurses to understand how people's attitudes and views affect their behaviours and care-seeking patterns. Examples of such nurse manager's sensitivity and adaptation included in the QLTE-PT are “I am open to realities that are different from mine” and “I adapt my leadership style according to the expectations, values, habits, beliefs, and cultures of the nursing team members.” The distinctiveness of this factor from other factors in the QLTE-PT is evident in its unique focus on leaders' ability to respect, understand, and adapt to cultural differences, as outlined by Matveev [47]. Transcultural leaders must demonstrate the ability to adapt to the distinct expectations of their organization, community, competitors, and clients [3]. This supports the factor's discriminant validity, as it captures unique aspects of transcultural leadership not covered by other factors.

Factor 5 “integration in the nursing work environment” comprises three items which highlight interventions targeted at the professional integration of immigrant nurses into the specificities of the nursing practice in the host unit, for example, “I implement a clinical orientation program specific to my unit to develop competencies of immigrant nurses with different levels of knowledge and professional experience” and “I recourse to international protocols to guide and standardise nursing practice in the team.” This factor demonstrated a good internal consistency (*α* = 0.76), indicating that the items are cohesively capturing the essence of immigrant nurses' integration in the work environment. Convergent validity is reinforced through literature emphasizing the need for supportive nursing work environments for diverse nursing staff. Integration in multicultural nursing work environments is a bidirectional process that involves efforts not only by the immigrant nurses but also by the host organization [48]. Rovito et al. [49] warn that sometimes nurses need to “unlearn” practices of nursing care that were common in their home country, since they are not a part of the nursing practice in the culture of the host country. According to Safari et al. [50], several strategies are needed to guide immigrant healthcare professionals in the culture of the host country before starting their clinical practice. A clinical orientation program is an example of a strategy that minimizes the impact of the challenges and difficulties migrant nurses experience, improves their well-being, the quality of care, and mitigates the risks to patient safety [51]. It is the nurse manager's responsibility to provide guidance, invest in multicultural education and mentoring for these nurses, guide their practice to provide safe and culturally congruent care, and ensure competence development and efficient use of their expertise [52]. Discriminant validity is evident, as this factor uniquely addresses the integration of immigrant nurses in the work environment, distinct from other transcultural nursing leadership characteristics evidenced in the other factors.

The last factor was labelled as “adjusting care to cultural expectations.” It demonstrated an acceptable internal consistency (*α* = 0.69), indicating that the items are able to capture nurse managers and leaders' interventions to meet patients' cultural expectations regarding nursing care. This factor is composed of three items such as “I request religious support congruent with patients' beliefs (e.g., Chaplain, Imam, Brahmin, Lama, and other spiritual leaders).” According to Leininger and McFarland [38], if patients receive nursing care that is not compatible and respectful with patients' ways of life, beliefs, and values, they will demonstrate signs of stress, nonadherence, cultural conflict, and ethical or moral concerns. This factor is in line with the guidelines for the implementation of culturally congruent nursing care in organizations, namely, the provision of structures and resources needed to meet patients' cultural needs [53]. It is also supported by existing literature on the responsibility of nurse managers to provide culturally and linguistically appropriate services to diverse populations [28, 54]. This literature supports the convergent validity of this factor. Furthermore, the distinctiveness of the factor, evident through discriminant validity, confirms its unique role in the QLTE-PT, differentiating it from other factors.

Although there is an overall agreement about the scenario of cultural diversity among patients and healthcare workers in Portugal, no studies have attempted to describe the leadership behaviours of nurse leaders and managers in multicultural nursing work environments in this country. Therefore, this paper is innovative and grants a questionnaire that addresses nurse leaders and managers' leadership behaviours in multicultural nursing work environments with applicability in Portugal.

### 4.1. Study Limitations

The findings of this study contribute to the field by providing a reliable and valid instrument for assessing transcultural nursing leadership. However, it is important to consider the limitations of this study and the implications they have for the generalizability and interpretation of the results.

First, self-report measures were utilized in this study, which are subject to potential biases. Participants may have provided socially desirable responses regarding their leadership behaviours in multicultural nursing work environments, leading to response bias. Efforts were made to minimize these biases through clear instructions to participants and ensuring data anonymity. However, it is important to acknowledge this limitation probability.

In addition, despite QLTE-PT having good internal consistency and construct validity, further validation in different cultural contexts is necessary. The current study focused on nurses registered with the Portuguese Order of Nurses and with experience of leadership in multicultural nursing work environments. Future research should aim to conduct cross-cultural validation to ensure the questionnaire's robustness across diverse cultural contexts. Future research should also include a criterion measure to provide a more comprehensive assessment of the questionnaire's validity.

## 5. Conclusions

This study successfully developed and validated the Portuguese Transcultural Nursing Leadership Questionnaire (QLTE-PT). The final instrument, comprising 23 items across six factors, demonstrates robust psychometric properties, including good internal consistency and construct validity. The QLTE-PT captures essential aspects of transcultural nursing leadership, making it a valuable tool for evaluating nurse managers and leaders' behaviours in multicultural nursing work environments. While acknowledging the study's limitations, the QLTE-PT represents a significant contribution to nursing management, offering insights into leadership behaviours in diverse healthcare settings. Due to its potential interest of use in other countries with multicultural healthcare settings, we recommend the development of guidelines with comprehensive information about different methodological approaches to support the decision-making and the quality of the cross-cultural adaptation process [55].

## 6. Implications for Nursing Management

The development and validation of the QLTE-PT hold important implications for nursing management and leadership. By providing a reliable and valid instrument to assess transcultural nursing leadership, the QLTE-PT offers nurse managers a valuable tool to gain insights into their own leadership behaviours in their multicultural units or organizations and acknowledges the needs of their nursing staff and patients from different cultural backgrounds. The questionnaire can assist nurse managers in identifying areas for improvement in their leadership and promotes a supportive work environment for their multicultural nursing staff, conducive to the well-being and engagement of nurses. It can also contribute to improving the quality of care provided to patients from different cultural backgrounds, ensuring patients' safety, satisfaction with nursing care, and higher levels of adherence to treatments.

## Figures and Tables

**Table 1:** Sociodemographic and professional characteristics (*n* = 463).

Sociodemographic and professional data		Frequency	Percentage
Education level	Bachelor	224	48.4
	Master degree	181	39.1
	Specialty	52	11.2
	Doctorate	6	1.3

Professional category	Nurse	172	37.1
	Nurse specialist	98	21.2
	Nurse manager	110	23.8
	Team leader	83	17.9

Work unit	Surgery	67	14.5
	Medicine	63	13.6
	Emergency room	56	12.1
	Paediatrics	47	10.2
	Community	38	8.2
	Outpatient clinics	22	4.8
	Psychiatry	13	2.8
	Obstetrics	8	1.7
	Others	149	32.2

Exposure to other cultures	Usually	220	47.5
	Sometimes	114	24.6
	Always	98	21.2
	Rarely	31	6.7

Cultural exposure context	Professional	182	39.3
	Personal	15	3.2
	Both	266	57.5

Training in multiculturalism	Yes	33	7.1
	No	430	92.9

International experience	Yes	99	21.4
	No	364	78.6

**Table 2:** Exploratory factor analysis of the QLTE-PT.

Items	Factors						Communalities
	1	2	3	4	5	6	
A35	0.784	—	—	—	—	—	0.698
A34	0.717	—	—	—	—	—	0.704
A32	0.711	—	—	—	—	—	0.638
A30	0.549	—	—	—	—	—	0.496
A31	0.533	—	—	—	—	—	0.596
A26	—	0.836	—	—	—	—	0.834
A27	—	0.765	—	—	—	—	0.727
A15	—	0.715	—	—	—	—	0.665
A28	—	0.566	—	—	—	—	0.641
A2	—	—	0.838	—	—	—	0.722
A3	—	—	0.730	—	—	—	0.787
A7	—	—	0.622	—	—	—	0.681
A24	—	—	0.532	—	—	—	0.608
A1	—	—	0.513	—	—	—	0.449
A5	—	—	—	0.833	—	—	0.781
A6	—	—	—	0.814	—	—	0.800
A8	—	—	—	0.635	—	—	0.738
A4	—	—	—	0.606	—	—	0.583
A25	—	—	—	0.521	—	—	0.519
A17	—	—	—	—	0.808	—	0.717
A13	—	—	—	—	0.561	—	0.602
A19	—	—	—	—	0.524	—	0.524
A38	—	—	—	—	—	0.791	0.791
A36	—	—	—	—	—	0.733	0.733
A37	—	—	—	—	—	0.564	0.564
Variance	13.9%	13.6%	11.3%	9.3%	8.8%	7.1%	
	64%						
Cronbach's alpha	0.81	0.79	0.76	0.76	0.76	0.69	
	0.90						

**Table 3:** Correlations and squared correlations between the factors.

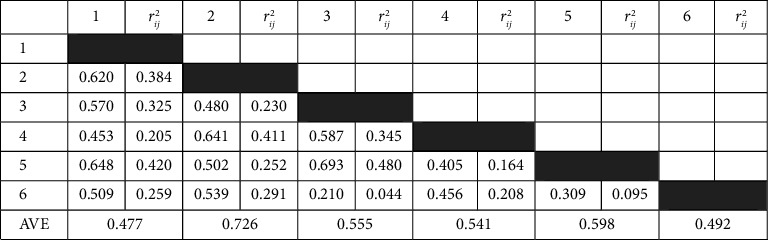

## Data Availability

The data used to support the findings of this study are included within the article.
